# Association Between High-Sensitivity C-Reactive Protein and Diabetic Kidney Disease in Patients With Type 2 Diabetes Mellitus

**DOI:** 10.3389/fendo.2022.885516

**Published:** 2022-06-16

**Authors:** Min Tang, Han Cao, Xiao-Hui Wei, Qin Zhen, Fang Liu, Yu-Fan Wang, Neng-Guang Fan, Yong-De Peng

**Affiliations:** ^1^ Department of Endocrinology and Metabolism, Shanghai General Hospital, Shanghai Jiao Tong University School of Medicine, Shanghai, China; ^2^ Shanghai General Hospital of Nanjing Medical University, Shanghai, China; ^3^ Department of Endocrinology, Songjiang District Central Hospital, Shanghai, China

**Keywords:** high-sensitivity C-reactive protein, diabetic kidney disease, type 2 diabetes mellitus, microvascular complications, inflammation

## Abstract

**Objective:**

High-sensitivity C-reactive protein (hs-CRP) is an inflammatory marker. This study aimed to identify the correlation between hs-CRP levels and diabetic kidney disease (DKD) in patients with type 2 diabetes mellitus (T2DM).

**Materials/Methods:**

This cross-sectional and observational study included 927 patients with T2DM. We collected the data of patients based on their medical data, including sociodemographic characteristics, concomitant diseases, laboratory results, and medical therapy. Multivariate logistic regression analysis was conducted to assess the relationship between hs-CRP levels and DKD. A restricted cubic spline (RCS) was used to assess the correlation of hs-CRP levels on a continuous scale with the DKD.

**Results:**

In total, 927 patients were recruited in our study. The median age of the recruited patients was 55 years, and there were 346 female patients and 581 male patients. The hs-CRP levels were evidently higher in patients with DKD than those without DKD. After adjusting for age, sex, diastolic blood pressure, systolic blood pressure, body mass index, neck circumference, waist circumference, hypertension, duration of diabetes, common carotid artery plaque, fasting plasma glucose, glycated hemoglobin, hemoglobin, erythrocyte, leukocyte, γ-glutamyl transferase, albumin, urea nitrogen, uric acid and triglyceride, a significant increase in the odds ratios (ORs) for DKD in the fourth hs-CRP quartile compared with the first quartile was observed (P value for trend= 0.003), and the ORs (95% confidence intervals) in the fourth quartile of hs-CRP were 1.968 (1.244–3.114) for DKD compared to the first quartile.. Moreover, the RCS curves presented a positive association between hs-CRP and DKD in total subjects, male subjects and female subjects, respectively.

**Conclusions:**

The results of our study indicated that hs-CRP levels were significantly and positively correlated with the presence of DKD, which may provide predictive and diagnostic values in clinical practice.

## Introduction

Diabetes mellitus (DM) has become a major and serious threat to global human health. In the population aged 20 to 79 years, approximately 536.6 million people were diagnosed with DM worldwide in 2021, and this number will rapidly grow to 783.2 million in 2045 worldwide (1). Type 2 DM (T2DM) has 90% proportion of DM, which is featured by insulin resistance and relatively insufficient insulin secretion (2). T2DM can lead to many chronic complications, one of which is microvascular complications. In addition, approximately one in four patients with T2DM will have comorbid diabetic kidney disease (DKD) in the progression of T2DM (3). DKD is the most common cause of leading to end-stage renal disease worldwide (4, 5) and is a primary cause of mortality among patients with DM (4). Therefore, early intervention plays a vital role in preventing DKD development. To establish effective intervention strategies for DKD in clinical practice, identifying risk factors related to the presence of DKD is available (6).

The pathogenesis of DKD is multifactorial, where chronic inflammation and immune response are considered the major factors (7, 8). High-sensitivity C-reactive protein (hs-CRP) is a systemic inflammation marker that has been revealed to be correlated with DKD development (9, 10). CRP is a reactant and sensitive marker in acute-phase of inflammation, and its level increases sharply during the process of inflammation and recovers to normal level when inflammation has ceased (11, 12). Although several studies have indicated that elevated CRP levels are found in DKD (6, 13), the evidence of relationship between CRP and DKD is insufficient, and related studies are limited.

Therefore, we conducted this cross-sectional and observational study that aimed to identify the correlation between hs-CRP levels and DKD in patients with T2DM.

## Materials and Methods

### Study Design

This study was a cross-sectional and observational one comprising 927 patients with T2DM, and all the subjects were recruited from the National Metabolic Management Center in Shanghai General Hospital (Songjiang district). The study was approved by the Ethics Committee of Shanghai General Hospital, Shanghai Jiao Tong University School of Medicine.

### Patient Data Collection

All the data analyzed in this study were extracted from the National Metabolic Management Center. In total, 927 patients with a prior diagnosis of T2DM were included in this study. The inclusion criteria of this study were as follows: with a prior diagnosis of T2DM, the diagnostic criteria for T2DM that were in accordance with the guidelines of the 1999 World Health Organization criteria (14), age ≥18 years. The exclusion criteria were as follows: presence of chronic nephritis, presence of immune deficiency syndrome, pregnancy, presence of malignant tumor, receiving chemotherapy, presence of active infection, presence of acute diabetic complications, and missing data.

### Study Variables

We collected data on patients from the National Metabolic Management Center based on their medical data, including sociodemographic characteristics, concomitant diseases, laboratory results, and medical therapy. The study variables were as follows: age; sex (male or female); educational level (under high school/high school or above); body mass index (BMI); diastolic blood pressure (DBP); systolic blood pressure (SBP); heart rate; head, neck, waist, and hip circumferences, duration of diabetes; hemoglobin, erythrocyte, leukocyte, albumin, urea nitrogen, creatinine, uric acid, triglyceride, total cholesterol, fasting plasma glucose (FPG), glycated hemoglobin (HbA1c), alanine transaminase, aspartate aminotransferase, γ-glutamyl transferase (γ-GT), high-density lipoprotein cholesterol (HDL-C), low-density lipoprotein cholesterol (LDL-C) levels, urinary albumin-to-creatinine ratio (UACR), hs-CRP levels and estimated glomerular infiltration rate (eGFR), hypertension (no/yes), hyperlipidemia (no/yes), and common carotid artery (CCA) plaque (no/yes).

### Definition of Variables

Hypertension was defined as SBP ≥ 140 mmHg and/or DBP ≥ 90 mmHg following repeated examination (15) or prior diagnosis of hypertension by a physician. Hyperlipidemia was defined as total cholesterol ≥ 240 mg/dL, triglycerides ≥ 200 mg/dL, LDL-C ≥ 160 mg/dL, and HDL-C < 40 mg/dL or prior diagnosis of hyperlipidemia by a physician. The scores of eGFR were computed by using the formula of Chronic Kidney Disease Epidemiology Collaboration (16). The UACR was calculated as the urinary albumin/creatinine ratio. DKD was defined as ACR ≥ 30 mg/g and/or eGFR < 60 mL/min/1.73 m^2^ (17).

### Statistical Analyses

Data are presented as numbers or medians (interquartile range). Continuous variables with skewed distribution were analyzed using Mann–Whitney U test, and categorical variables were evaluated using the χ^2^ to identify intergroup differences. Multivariate logistic regression models were used to identify the independent effects of hs-CRP level on the presence of DKD. In the univariate analysis, variables with P value < 0.05 were enrolled in the multivariate logistic regression model. A two-sided P-value < 0.05 was regarded as that the difference has statistical significance between the two groups.

Moreover, multivariate logistic regression analysis was conducted to assess the relationship between hs-CRP levels and DKD, and the odds ratios (ORs) and 95% confidence intervals (CIs) were calculated. Model 1 was adjusted for age and sex. Model 2 was further adjusted for DBP, SBP, BMI, neck circumference, and waist circumference. Model 3 was further adjusted for hypertension, duration of diabetes, and CCA plaque. Model 4 was further adjusted for FPG, HbA1c, hemoglobin, erythrocyte, leukocyte, γ-GT, albumin, urea nitrogen, uric acid, and triglyceride.

The restricted cubic spline (RCS) with four knots at the 5th, 35th, 65th, and 95th percentiles was executed on a continuous scale with the presence of DKD. Furthermore, the associations between hs-CRP and DKD in different subgroups by grouping age, sex, BMI, hypertension, CCA plaque and HbA1c were identified by multivariate logistic regression models, and the ORs and 95% CI were calculated. In addition, the interaction between these subgroup variables and hs-CRP were calculated. All statistical analyses in our study were executed by IBM SPSS (version 25.0) and R statistical software (version 4.0.5).

## Results

### Characteristics of Study Subjects

A total of 927 patients were enrolled in our study. The median age of the recruited patients including 346 female patients and 581 male patients was 55 years, and there were 322 patients with DKD and 605 patients without DKD in our study. The characteristics of the patients with and without DKD were summarized in **Table 1**. Age, sex, DBP, SBP, BMI, neck circumference, waist circumference, hypertension, duration of diabetes, CCA plaque, FPG, HbA1c, hemoglobin, erythrocyte, leukocyte, γ-GT, albumin, urea nitrogen, uric acid, triglyceride, hs-CRP, UACR and eGFR were significantly different between the two groups.

**Table 1:** Characteristics of the study subjects.

Variables	No DKD (n = 605; 65.26%)	DKD (n = 322; 34.74%)	P value
Age (years)	54 (42-63)	55 (46-65)	0.017
DBP (mmHg)	76 (67-80)	78 (70-84)	<0.001
SBP (mmHg)	120 (114-130)	128(120-140)	<0.001
Heart rate (beats per minute)	78 (75-86)	78 (76-88)	0.072
BMI (kg/m^2^)	24.96 (23.00-27.35)	25.80 (23.70-28.70)	<0.001
Head circumference (cm)	54 (52-56)	54 (52-56)	0.272
Neck circumference (cm)	39 (36-41)	40 (37-42)	0.007
Waist circumference (cm)	92 (85-97)	93 (88-100)	0.001
Hip circumference (cm)	96 (93-100)	96 (93-101)	0.106
Duration of diabetes (months)	61 (1-141)	98 (12-171)	0.001
FPG (mmol/L)	6.91 (5.62-8.53)	7.67 (5.98-9.48)	<0.001
HbA1c (%)	8.50 (7.20-10.45)	9.25 (7.50-11.00)	0.002
Hemoglobin (g/L)	145 (132-154)	137 (124-149)	<0.001
Erythrocyte (×10^12^/L)	4.75 (4.38-5.05)	4.53 (4.14-4.92)	<0.001
Leukocyte (×10^9^/L)	5.99 (5.09-7.07)	6.54 (5.49-7.75)	<0.001
ALT (IU/L)	20 (14-35)	20 (14-30)	0.305
AST (IU/L)	18 (15-25)	18 (15-25)	0.365
γ-GT (IU/L)	24 (16-40)	27 (18-41)	0.003
Albumin (g/L)	43.2 (40.8-45.5)	42.3 (39.3-45.0)	0.001
Urea nitrogen (mmol/L)	5.22 (4.15-6.86)	5.72 (4.45-7.70)	0.007
Creatinine (μmol/L)	58.4 (47.8-67.5)	59.4 (45.4-80.6)	0.134
Uric acid (μmol/L)	317.0 (256.5-376.0)	328.6 (277.0-401.3)	0.002
Triglyceride (mmol/L)	1.58 (1.14-2.27)	1.87 (1.40-2.77)	<0.001
Total cholesterol (mmol/L)	4.56 (3.71-5.23)	4.69 (3.83-5.49)	0.060
HDL-C (mmol/L)	0.91 (0.79-1.09)	0.90 (0.78-1.05)	0.366
LDL-C (mmol/L)	2.60 (1.91-3.17)	2.57 (1.92-3.33)	0.734
Hs-CRP (mg/L)	1.2 (0.5-2.6)	1.9 (0.8-4.5)	<0.001
UACR (μg/mg)	12.99 (8.79-19.02)	74.87 (41.88-244.78)	<0.001
eGFR (mL/min per 1.73 m^2^)	111.00 (101.15-122.95)	105.68 (87.21-119.38)	<0.001
Sex(male/female)	402/203	179/143	0.001
Educational level (under high school/high school or above)	305/300	143/179	0.082
Hypertension(no/yes)	361/244	118/204	<0.001
Hyperlipidemia(no/yes)	454/151	225/97	0.091
CCA plaque(no/yes)	344/261	147/175	0.001

Data were numbers or median (interquartile range). Continuous variables used Mann-Whitney U test and categorical variables used chi-squared test for comparing the baseline characteristics of patients with diabetic kidney disease and without diabetic kidney disease.

DBP, diastolic blood pressure; SBP, systolic blood pressure; BMI, body mass index; FPG, fasting plasma glucose; HbA1c, glycated hemoglobin; ALT, alanine transaminase; AST, aspartate aminotransferase; γ-GT, γ-glutamyl transferase; HDL-C - high-density lipoprotein cholesterol; LDL-C - low-density lipoprotein cholesterol; Hs - CRP, high-sensitivity C-reactive protein; UACR, urinary albumin to creatinine ratio; eGFR, estimated glomerular infiltration rate; CCA plaque, common carotid artery plaque; DKD, diabetic kidney disease.

### Association Between hs-CRP Levels and DKD

**Table 2** demonstrates that increased hs-CRP was correlated with higher odds of DKD after adjusting for several confounding factors. In our study, a significant increase in the ORs for DKD from the first to the fourth hs-CRP quartiles in the study subjects was observed after adjusting for age, sex, DBP, SBP, BMI, neck circumference, waist circumference, hypertension, duration of diabetes, and CCA plaque in the multivariate regression model (P value for trend< 0.001). When the multivariate regression model was further adjusted for FPG, HbA1c, hemoglobin, erythrocyte, leukocyte, γ-GT, albumin, urea nitrogen, uric acid and triglyceride, a similar increase in ORs for DKD from the first to the fourth hs-CRP quartiles was still observed (P value for trend = 0.003). Compared with the lowest quartile, the ORs (95% CIs) of the fourth quartile for DKD were 1.968 (95% CI, 1.244–3.114) after adjusting for age, sex, DBP, SBP, BMI, neck circumference, waist circumference, hypertension, duration of diabetes, CCA plaque, and FPG, HbA1c, hemoglobin, erythrocyte, leukocyte, γ-GT, albumin, urea nitrogen, uric acid and triglyceride. 

**Table 2:** Relations of high-sensitivity C-reactive protein with diabetic kidney disease in patients with type 2 diabetes mellitus.

hs-CRP, mg/L					P value for trend
	Q1 (n=246)	Q2 (n=229)	Q3 (n=223)	Q4 (n=229)	
Model 1 - OR (95% CI)	Ref.	1.249 (0.832–1.875)	1.718 (1.148–2.572)	2.839 (1.914–4.210)	<0.001
Model 2 - OR (95% CI)	Ref.	1.153 (0.757–1.756)	1.509 (0.988–2.305)	2.472 (1.633–3.743)	<0.001
Model 3 - OR (95% CI)	Ref.	1.288 (0.836–1.984)	1.568 (1.017–2.419)	2.767 (1.805–4.243)	<0.001
Model 4 - OR (95% CI)	Ref.	1.169 (0.741–1.843)	1.436 (0.909–2.270)	1.968 (1.244–3.114)	0.003

Model 1 was adjusted for age, sex. Model 2 was further adjusted for diastolic blood pressure, systolic blood pressure, body mass index, neck circumference and waist circumference. Model 3 was further adjusted for hypertension, duration of diabetes and common carotid artery plaque. Model 4 was further adjusted for fasting plasma glucose, glycated hemoglobin, hemoglobin, erythrocyte, leukocyte, γ-glutamyl transferase, albumin, urea nitrogen, uric acid and triglyceride. The quartile ranges of Q1, Q2, Q3, and Q4 of hs-CRP level were < 0.6, 0.6-1.4, 1.4-3.1,> 3.1 mg/L, respectively. Q1 is the reference group. Multivariate logistic regression analyses were performed to estimate the ORs and corresponding 95% CIs for diabetic kidney disease.

Q, quartile, OR, odds ratio, CI, confidence interval, hs - CRP, high-sensitivity C-reactive protein.

**Figure 1:**
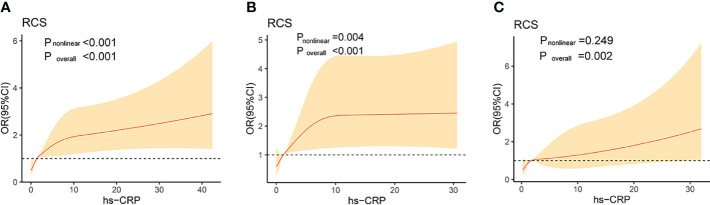
Association of high-sensitivity C-reactive protein levels on a continuous scale and diabetic kidney disease in total subjects **(A)**, male subjects **(B)** and female subjects **(C)**. The solid lines represent the odds ratios and the shaded areas represent the 95% confidence intervals. Model was adjusted for age, body mass index, neck circumference,waist circumference, hypertension, duration of diabetes, common carotid artery plaque, fasting plasma glucose, glycated hemoglobin. OR, odds ratio; CI, confidence interval; RCS, restricted cubic splines; hs - CRP, high-sensitivity C-reactive protein.

In our study, we further executed the RCS to flexibly model the correlation of hs-CRP on a continuous scale with DKD in total subjects, male subjects and female subjects, respectively. The RCS curves showed a positive association in total subjects (P for non-linearity <0.001), male subjects (P for non-linearity =0.004) and female subjects (P for non-linearity =0.249), respectively (**Figure 1**).

### Subgroup Analyses

**Figure 2:**
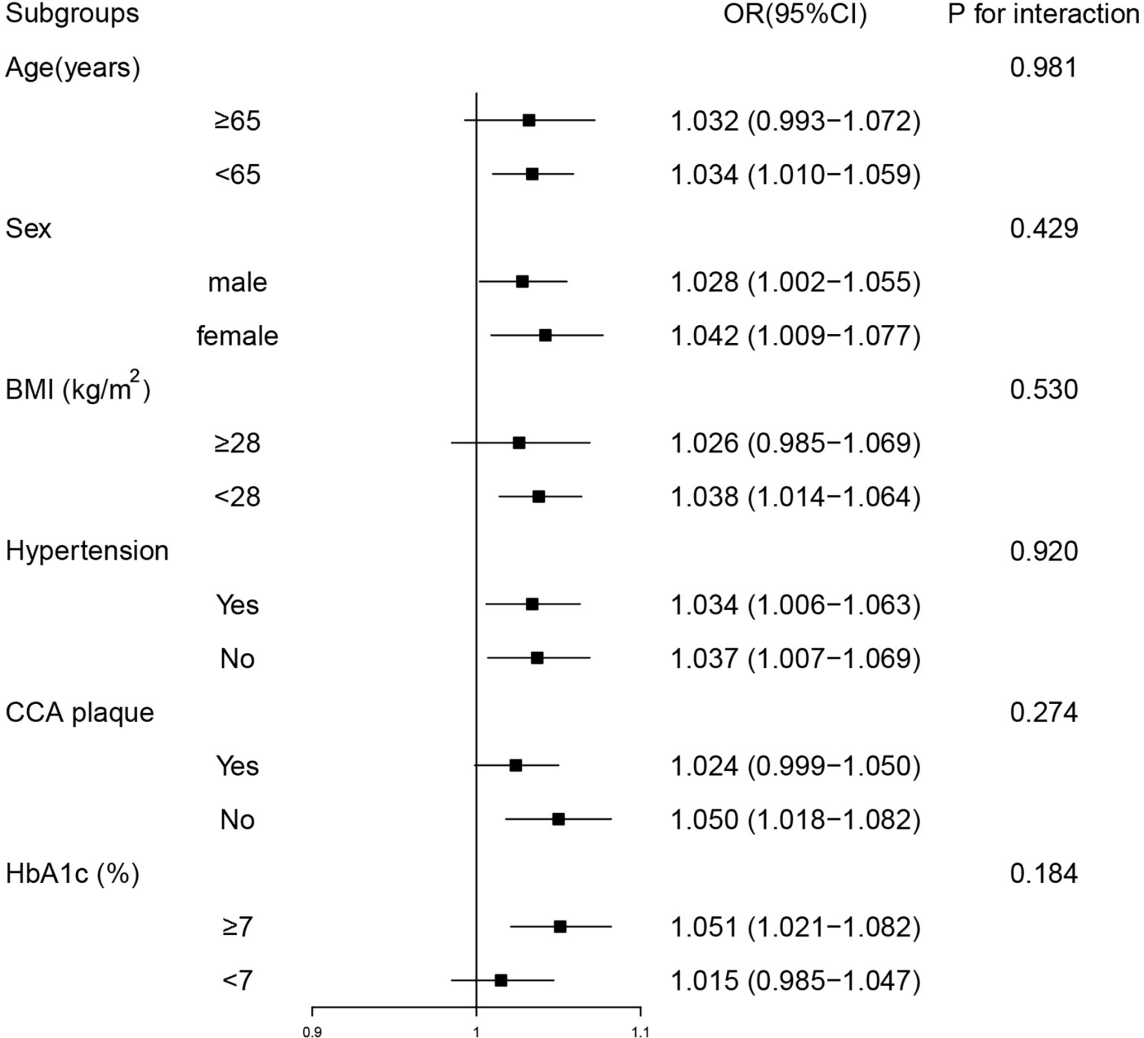
Stratified analyses of the associations between high-sensitivity C-reactive protein levels and diabetic kidney disease. Model was adjusted for age, sex, body mass index, neck circumference,waist circumference, hypertension, duration of diabetes, common carotid artery plaque, fasting plasma glucose, glycated hemoglobin. Subgroup variable was excluded from the model. OR, odds ratio; CI, confidence interval; BMI, body mass index; CCA plaque, common carotid artery plaque; HbA1c, glycated hemoglobin.

Subgroup analyses were performed to determinate the potential effect modifiers, and subjects were stratified by age (< 65 or ≥ 65 years), sex (male or female), BMI (< 28 or ≥ 28 kg/m^2^), hypertension (no or yes), CCA plaque (no or yes) and HbA1c (< 7 or ≥ 7%), respectively. The results of subgroup analyses showed that hs-CRP was positively correlated with DKD in most categories. No significant interaction was found between hs-CRP level and age, sex, BMI, hypertension, CCA plaque, or HbA1c, respectively (**Figure 2**).

## Discussion

In this study, we identified the correlation between hs-CRP levels and DKD in patients with T2DM. We found that hs-CRP levels were evidently higher in subjects with DKD when compared with subjects with non-DKD. In addition, from the lowest quartile to highest quartile of hs-CRP levels, the ORs for DKD were significantly increase. Moreover, our results indicated a significant positive correlation between hs-CRP levels and DKD in the study subjects, which may provide scientific evidence for further basic and clinical research.

CRP levels are elevated in acute-phase inflammation. A previous study suggested that the normal population with elevated CRP levels have a higher risk of T2DM (18), and accumulated evidence suggests that circulating CRP levels have predictive value for the incidence of DM (18–20). Moreover, CRP levels were correlated with endothelial dysfunction among DM populations, and CRP can predict DM complications (19). In our study, we found a significantly positive correlation between hs-CRP levels and the presence of DKD in patients with T2DM, which is consistent with several published studies (10, 21, 22). However, the controversial view proposed by a previous study was that no correlation was found between hs-CRP levels and diabetic vascular complications (23), which may be inadequate for providing adequate evidence because of the limitations of the small sample size. A meta-analysis was executed to explore the correlation between the concentration of hs-CRP and DKD, and it was verified that elevated hs-CRP levels were correlated with the prevalence of DKD, and that hs-CRP levels can be a predictor of DKD in patients with DM (10). In addition, in the results of the RCS curves, although there was a significant positive correlation between hs-CRP and DKD after adjusting confounders, a difference was observed in female and male subjects with increasing hs-CRP levels, which may provide scientific evidence for further research.

The pathogenesis of DKD is multifactorial, and analysis studies of the genome-wide transcriptome have shown that there is a strong prevalence of inflammatory signaling pathways in DKD (24). Inflammation in the kidney activated by mononuclear phagocytic lineage cells is one of the most significant biological mechanisms underlying DKD (25–27), and several inflammatory cytokines, such as interleukin (IL)-1, IL-6, IL-18, tumor necrosis factor-α (TNF-α) and CRP, may lead to the pathogenesis of DKD (9, 28–32). As CRP is related with insulin resistance and hyperglycemia, higher CRP levels are correlated with the development of DKD by aggravating glycemic control in DM populations (33). Previous studies have indicated that the nuclear transcription factor-kappa B (NF-κB) signaling hs-CRP pathway is activated in DKD, and hs-CRP regulates many proinflammatory cytokines through the NF-κB signaling pathway (34, 35). Moreover, hs-CRP can also be induced by hyperglycemia, which accelerates inflammation in the kidneys (10). Therefore, hs-CRP level can serve as a predictor of DKD in T2DM populations.

This study has some limitations. First, the main limitation was its cross-sectional and observational nature, and longitudinal studies are necessary to test the association over time, and future prospective studies are necessary. Second, the sample size was small because all the data of this study were from a single hospital; therefore, the quality of the results may be negatively affected. Further studies with a larger population are required to examine this association. Third, our study did not explore the underlying mechanisms of hs-CRP and DKD, and whether high hs-CRP levels are a cause or result of DKD should be clarified. In addition, our study did not include several inflammatory cytokines, such as IL-6, IL-18, and TNF-α, which are correlated with DKD, which may be potential confounding factors affecting the results.

## Conclusion

In our study, we found that hs-CRP levels were significantly higher in study subjects with DKD than in the subjects with non-DKD, and hs-CRP levels have been proven to be correlated with the presence of DKD in T2DM patients, which might provide predictive and diagnostic values in clinical practice.

## Data Availability Statement

The raw data supporting the conclusions of this article will be made available by the authors, without undue reservation.

## Ethics Statement

The studies involving human participants were reviewed and approved by Ethics Committee of Shanghai General Hospital, Shanghai Jiao Tong University School of Medicine. Written informed consent was obtained from all participants.

## Author Contributions

Study design: N-GF and Y-DP; Collection and assembly of data: MT and HC; Data analysis: MT and HC; data interpretation: N-GF and Y-DP; Manuscript writing: All authors; final approval of manuscript: All authors.

## Funding

This study was supported by the National Natural Science Foundation of China (81870596, 81400785) and the Natural Science Foundation of Shanghai (21ZR1451200).

## Conflict of Interest

The authors declare that the research was conducted in the absence of any commercial or financial relationships that could be construed as a potential conflict of interest.

## Publisher’s Note

All claims expressed in this article are solely those of the authors and do not necessarily represent those of their affiliated organizations, or those of the publisher, the editors and the reviewers. Any product that may be evaluated in this article, or claim that may be made by its manufacturer, is not guaranteed or endorsed by the publisher.
